# Predictable modulation of cancer treatment outcomes by the gut microbiota

**DOI:** 10.1186/s40168-020-00811-2

**Published:** 2020-03-05

**Authors:** Yoshitaro Heshiki, Ruben Vazquez-Uribe, Jin Li, Yueqiong Ni, Scott Quainoo, Lejla Imamovic, Jun Li, Maria Sørensen, Billy K. C. Chow, Glen J. Weiss, Aimin Xu, Morten O. A. Sommer, Gianni Panagiotou

**Affiliations:** 1 Systems Biology & Bioinformatics Unit, Leibniz Institute for Natural Product Research and Infection Biology, Hans Knöll Institute, Jena, Germany; 2 Systems Biology and Bioinformatics Group, School of Biological Sciences, Faculty of Sciences, The University of Hong Kong, Hong Kong, China; 3 Novo Nordisk Foundation Center for Biosustainability, Technical University of Denmark, 2800 Kgs. Lyngby, Denmark; 4 State Key Laboratory of Pharmaceutical Biotechnology, University of Hong Kong, Hong Kong, China; 5 Department of Medicine, Li Ka Shing Faculty of Medicine, University of Hong Kong, Hong Kong, China; 6 Department of Infectious Diseases and Public Health, The Jockey Club College of Veterinary Medicine and Life Sciences, City University of Hong Kong, Hong Kong, China; 7 School of Data Science, City University of Hong Kong, Hong Kong, China; 8 School of Biological Sciences, Faculty of Sciences, The University of Hong Kong, Hong Kong, China; 9 University of Arizona College of Medicine-Phoenix, Phoenix, AZ USA; 10 Department of Microbiology, Li Ka Shing Faculty of Medicine, The University of Hong Kong, Hong Kong, China

**Keywords:** Gut microbiota, Cancer, Treatment outcome, Machine learning

## Abstract

The gut microbiota has the potential to influence the efficacy of cancer therapy. Here, we investigated the contribution of the intestinal microbiome on treatment outcomes in a heterogeneous cohort that included multiple cancer types to identify microbes with a global impact on immune response. Human gut metagenomic analysis revealed that responder patients had significantly higher microbial diversity and different microbiota compositions compared to non-responders. A machine-learning model was developed and validated in an independent cohort to predict treatment outcomes based on gut microbiota composition and functional repertoires of responders and non-responders. Specific species, *Bacteroides ovatus* and *Bacteroides xylanisolvens*, were positively correlated with treatment outcomes. Oral gavage of these responder bacteria significantly increased the efficacy of erlotinib and induced the expression of CXCL9 and IFN-γ in a murine lung cancer model. These data suggest a predictable impact of specific constituents of the microbiota on tumor growth and cancer treatment outcomes with implications for both prognosis and therapy.

## Background

Cancer is one of the leading causes of mortality worldwide, with nearly one in six deaths globally attributed to cancer [1]. Among several treatment options, chemotherapy and immunotherapy are applied to treat cancer by preventing cancer cell division or boosting the immune system to eliminate cancerous cells [2]. In spite of recent progress, treatment outcomes are still unsatisfactory for most cancer types. The gut microbiota is increasingly considered an important factor associated with both tumor development and the efficacy of anticancer therapies [3]. Specific gut bacteria have been shown to affect cancer treatments through direct drug metabolism and modulation of the host immune response [4]. Bacterial beta-glucuronidase can convert irinotecan, an anti-cancer chemotherapy drug, to a toxic metabolite [5], and intratumor bacterial cytidine deaminase can degrade gemcitabine with a direct impact on treatment outcomes [6]. The gut microbiota or defined synthetic communities can also impact treatment outcomes through immune modulation mechanisms such as regulating T cell differentiation [7–9]. Indeed, the gut microbiota can substantially impact immune checkpoint inhibitor therapy [10–13] and antibiotic use is associated with poor treatment outcomes with checkpoint inhibitors [14].

Previous studies have focused on elucidating the role of individual microbes or communities in a specific cancer type and therapeutic intervention. In the present study, we investigated the role of gut microbiota in a cancer patient cohort that included eight different cancer types treated with either cytotoxic or targeted chemotherapy, immunotherapy, or a combination. Our objective here was to demonstrate a more global finding of a microbiota signature that is independent of cancer type and heterogeneity. Using a combination of human feces shotgun metagenomic sequencing, in vitro and in vivo mouse models, we found that cancer treatment outcomes in this diverse cohort can be substantially modulated by the abundances of specific gut bacteria, supporting a recent study in healthy individuals to identify general activators of the immune system [15].

## Results

### Limited impact of cancer therapy on individual gut microbiota

Our cohort was comprised of 26 cancer patients of various cancer types, treated either with cytotoxic or targeted chemotherapy (*n* = 15) or a combination of cytotoxic or targeted chemotherapy with immunotherapy (*n* = 11) (Table S1). We collected 71 fecal samples from the 26 patients at four different time points (B1: *baseline*, B2: *baseline at least 24 h after B1*, T1: *end of cycle 1 of treatment* ± *5 days*, T2: *end of cycle 2 of treatment* ± *5 days*). All the samples were further combined into two groups, namely *baseline* (*n* = 31, comprised of B1 and B2) and *treatment* (*n* = 40, comprised of T1 and T2).

**Fig. 1:**
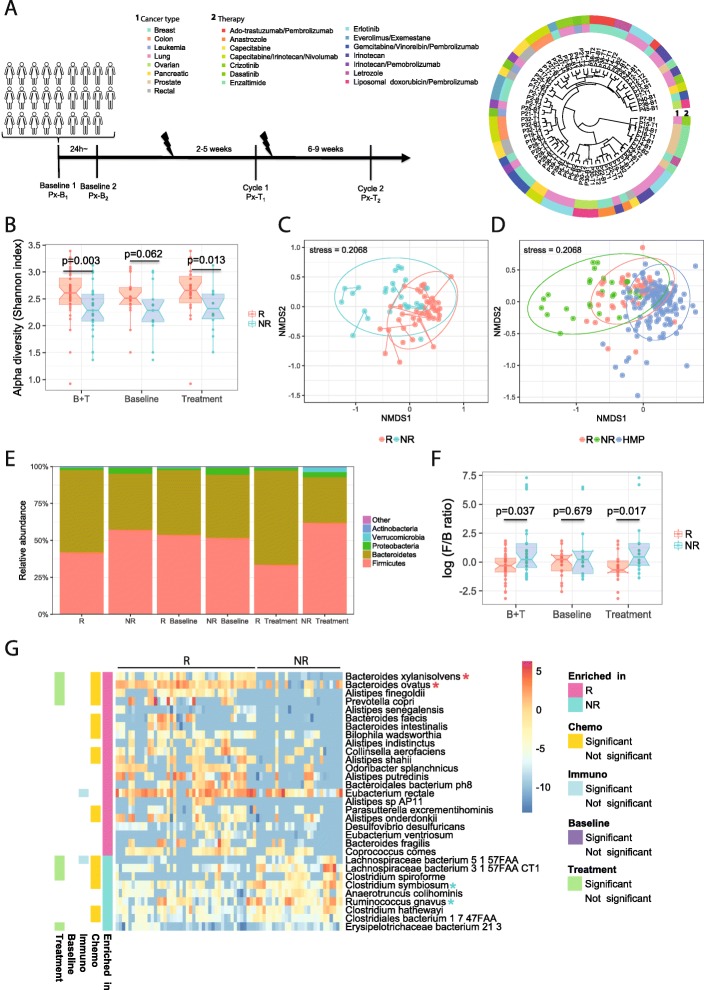
Taxonomic analysis of intestinal microbiota of cancer patients. **a** Sample collection scheme and dendrogram based on Bray-Curtis dissimilarity. **b** Alpha diversity (Shannon index) of the gut microbiota in *responders* (R) and *non-responders* (NR). **c** Non-metric multidimensional scaling (NMDS) plot of *R* and *NR* in human cancer samples based on the gut microbial compositions using Bray-Curtis dissimilarities (ANOSIM *p* = 0.0001). Intrapatient samples are linked to each other. **d** NMDS plot of *R*, *NR*, and HMP samples based on the gut microbial compositions at the species level using Bray-Curtis dissimilarities (ANOSIM *p* = 0.0001). **e** Phylogenetic composition of cancer samples at the phylum level. **f***Firmicutes*/*Bacteroidetes* (F/B) ratio of cancer samples. **g** Heatmap of differentially abundant species detected in the comparison of *R* and *NR* (FDR *p* < 0.05, Wilcoxon rank-sum test). R-associated and NR-associated bacteria validated in mouse model are shown in red and cyan asterisks, respectively

We assessed the structure of the gut microbiome in all available samples (*n* = 71) via shotgun metagenomic sequencing, generating 6.1 Gbp of sequencing data on average (s.d. 1.3 Gbp per sample) (Table S2). The taxonomic profiling revealed that *Bacteroidetes* (44.51% on average) and *Firmicutes* (44.04%) were the most abundant phyla across all samples, followed by *Proteobacteria* (4.09%) and *Verrucomicrobia* (3.53%). To test whether the gut microbiota compositions of patients with different cancer types share similar profiles, we investigated the cancer type-specific microbiome signatures. The 26 patients were classified according to their primary site of tumors: lung (*n* = 8), breast (*n* = 7), colon (*n* = 2), rectal (*n* = 2), pancreatic (*n* = 2), ovarian (*n* = 2), prostate (*n* = 2), and blood (*n* = 1). The dendrogram clustering based on taxonomic profiles showed that interpatient samples with the same cancer type did not necessarily cluster together, while the intrapatient samples tend to cluster closely with relatively minimal impact from the anticancer treatment (Fig. 1a and Fig. S1A) as previously reported [16–18]. Subsequently, we further compared the gut microbiota communities of *baseline* versus *treatment* to investigate any global patterns of anticancer therapies on gut microbial compositions. The alpha diversity comparison indicated that the *baseline* and *treatment* samples had similar levels of diversity (*p* = 0.265, Wilcoxon rank-sum test) (Fig. S2). Likewise, the ordination plot based on the beta diversity (Bray-Curtis dissimilarity) indicated no difference between *baseline* and *treatment* (*p* = 0.364, ANOSIM) (Fig. S1B), suggesting that anticancer therapy may not introduce drastic changes to the overall structure of the gut microbial community. Moreover, no differentially abundant taxa, functional pathways, or modules could be identified by comparing *baseline* versus *treatment* samples in our data set.

Given the well-reported stability and resilience of individual signatures of human gut microbiota [17, 18], as well as the limited and non-significant effects of cancer types and anticancer treatments observed in our cohort, we combined the 71 samples and, similarly to microbiome meta-analysis studies [15, 19], performed a comparison with publicly available data to evaluate whether the cancer patients present distinct gut microbial profiles. We used, in the comparison, the gut microbiome samples of 138 healthy individuals from the Human Microbiome Project (HMP) [16], which, as our cohort, also consists of US subjects. The beta diversity comparison of cancer and HMP microbiome samples revealed that the two cohorts had significantly different species compositions of intestinal bacteria (*p* = 0.0001, ANOSIM) (Fig. S3A), while there was no significant difference on alpha diversity at the species level between the two cohorts (*p* = 0.07373, Wilcoxon rank-sum test) (Fig. S3B). In HMP, the mean abundance of the phylum *Bacteroidetes* across all HMP stool samples was 74.96%, followed by 22.07% of *Firmicutes*, indicating that the cancer cohort had a significantly higher *Firmicutes*/*Bacteroidetes* (F/B) ratio (*p* = 2.461e−13, Wilcoxon rank-sum test) (Fig. S3C). Compared with healthy individuals, a higher F/B ratio has also been observed in patients with irritable bowel syndrome (IBS), hypertension, autism, and chronic fatigue syndrome in case control studies [20–23]. Taken together, these comparisons above suggest that cancer treatments may not significantly disrupt the patients’ individual signatures of gut microbiota; however, the cancer patients have distinct gut microbiota features compared to the healthy cohort.

#### *Responders* have higher ecological diversity than *non-responders*

To evaluate the association between the microbial community and treatment outcome, we grouped the patients based on their response to treatment (*responders*: *R*, *n* = 16; *non-responders*: *NR*, *n* = 10). The classification of patients was based on the Response Evaluation Criteria in Solid Tumors (RECIST 1.1) [24] or immune-related response criteria (iRECIST) [25]. The *R* group achieved a favorable response (complete or partial response or stable disease status) as their best response, while the *NR* group showed disease progression as their best response to the administered systemic treatment. The patients in the two groups were similar in terms of stage of cancer, sex, age, and therapy type (Table S3). A comparison of the gut microbiome of these two groups revealed that *R* had higher alpha diversity than *NR* (*p* = 0.003, Wilcoxon rank-sum test, combined samples from *baseline* and *treatment*) (Fig. 1b). It led to the same conclusion when using just *treatment* samples (*p* = 0.008, Wilcoxon rank-sum test), though only showed trends when focusing on the *baseline* samples. Despite the difference in alpha diversity, *R* and *NR* showed similar levels of species richness (Chao1) (*p* = 0.674, Wilcoxon rank-sum test) (Fig. S4). Furthermore, the ordination plot based on Bray-Curtis dissimilarities revealed distinct intestinal microbial compositions at the species level between *R* and *NR* (*p* = 0.0001, ANOSIM) (Fig. 1c). Unweighted and weighted UniFrac distances were consistent with this result (*p* = 0.0001 and *p* = 0.0006). Interestingly, we also observed a clear gradation of *NR*, *R*, and healthy subjects (HMP) (*p* = 0.0001, ANOSIM) (Fig. 1d), with the majority of *R* samples overlapping with the HMP subjects, whereas *NR* samples were clearly distinct from those of the healthy subjects. This gradation suggests that the patients in *R* group have relatively more similar gut microbiota profiles to the healthy individuals.

No significant differences of alpha diversity between the *baseline* and *treatment* were observed either in *R* or *NR* (*p* = 0.3254 and *p* = 0.616 for *R* and *NR*, respectively, Wilcoxon rank-sum test) (Fig. 1b). Furthermore, the treatment impact on the gut microbiota of the two groups (*R* and *NR*) was also measured based on the Bray-Curtis dissimilarities between intrapatient *baseline* and *treatment* using the relative abundances of species or strains. The comparison showed no difference between *R* and *NR* in terms of the therapy impact on their gut microbial compositions at the community level (*p* = 0.216 and *p* = 0.204 for species and strains, respectively, Wilcoxon rank-sum test) (Fig. S5).

### Identification of specific taxa related to cancer treatment response

We next searched for differentially abundant taxa in the gut microbiome of *R* versus *NR*. The enrichment analysis revealed that, at the phylum level, *Bacteroidetes* was enriched in *R* in the treatment samples (FDR *p* = 0.031, Wilcoxon rank-sum test) but not in the baseline samples (FDR *p* = 0.540, Wilcoxon rank-sum test) (Fig. 1e). Additionally, comparing *Firmicutes*/*Bacteroidetes* (F/B) ratios, we noticed that *NR* showed a significantly higher ratio than *R* (*p* = 0.037, Wilcoxon rank-sum test) (Fig. 1f) and healthy individuals from the HMP (138 subjects, *p* = 1.617e−09, Wilcoxon rank-sum test), which is in agreement with the findings described above regarding the microbiome profiles of healthy individuals and cancer patients.

In the comparison between *R* and *NR*, 31 differentially abundant species (FDR *p* < 0.05, Wilcoxon rank-sum test) were identified. As shown in Fig. 1g, 22 and 9 species were *R*-*enriched* and *NR-enriched*, respectively. *Bacteroides xylanisolvens*, *Bacteroides ovatus*, *Prevotella copri*, and seven *Alistipes* species, among others, were found to be significantly enriched in *R* compared to *NR* (FDR *p* < 0.05, Wilcoxon rank-sum test) (Fig. 1g). We found that ~ 73% (16/22) of these species are classified at the phylum level as *Bacteroidetes*. In contrast, all 9 *NR-enriched* species, including *Clostridium symbiosum* and *Ruminococcus gnavus*, were classified as *Firmicutes* at the phylum level.

**Fig. 2:**
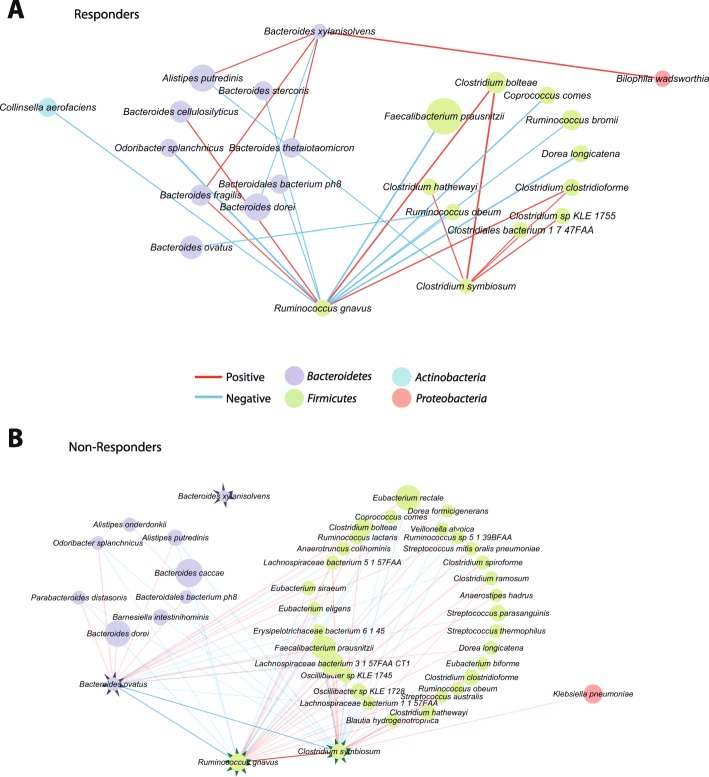
Bacterial species co-abundance networks. **a** Network in *responders*. **b** Network in *non*-*responders*. Each node represents a species and edges correspond to significant species-species associations as inferred by BAnOCC [26]. The size of each node is proportional to the mean relative abundance. The 95% credible interval criteria were used to assess significance, and estimated correlations were then filtered with the correlation coefficient ≥ 0.4. The shown subnetworks were made by extracting the edges that are connected with *B*. *ovatus*, *B*. *xylanisolvens*, *C*. *symbiosum*, and *R*. *gnavus*, which are further highlighted

Next, we reconstructed the species co-abundance networks separately for *R* and *NR* using BAnOCC [26]. The *R* network showed that *B. xylanisolvens* was correlated with other *Bacteroidetes* species and *Proteobacteria*, while this species did not show any significant associations in the *NR* network (Fig. 2a). On the other hand, the *NR* network shows that *C. symbiosum* and *R. gnavus* have a positive association with each other and both have a negative association with one of the *R-associated* species *B. ovatus* (Fig. 2b). Furthermore, in the *NR* network, both *C. symbiosum* and *R. gnavus* retained their positive interactions mostly within *Firmicutes* with only one exception (a positive interaction between *C. symbiosum* and *Klebsiella pneumoniae*), whereas their interactions with *Bacteroidetes* species were all negative. Altogether, it is suggested that the high abundances of *C*. *symbiosum* and *R*. *gnavus* in *NR* might promote the dominance of *Firmicutes* and impede *Bacteroidetes* by their intra-phylum positive associations along with the negative associations with *Bacteroidetes* species including *B. ovatus*. This observation is in line with the aforementioned high *Firmicutes*/*Bacteroidetes* (F/B) ratio in *NR* (Fig. 1f). Lastly, *R. gnavus*, as well as other *Firmicutes* species, were positively correlated with the F/B ratio (*r* = 0.5665, *p* = 0.0021, Pearson correlation) (Fig. S6).

### Anabolism enriched in *responders*’ and catabolism in *non-responders*’ microbial communities

**Fig. 3:**
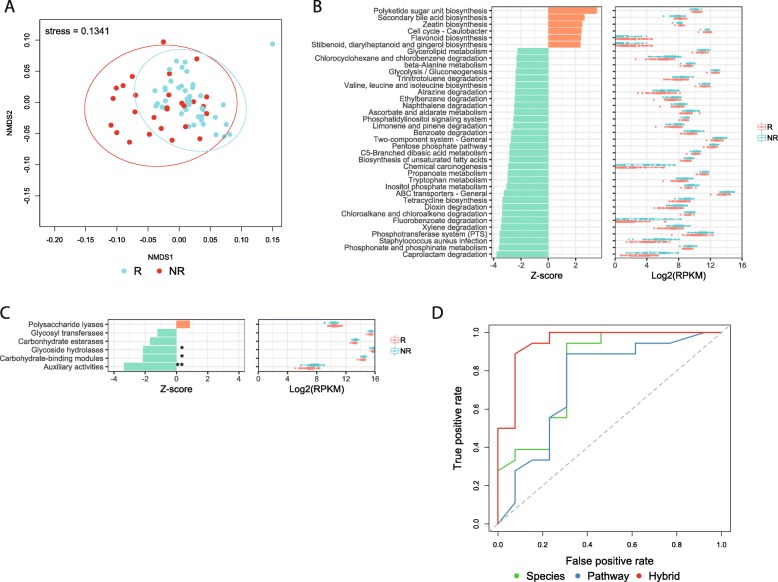
Functional profiles of intestinal microbiota of cancer patients. **a** NMDS plot of cancer samples based on KEGG pathway abundances using Bray-Curtis dissimilarities (ANOSIM *p* = 0.0299). **b** Differentially abundant KEGG pathways (FDR *p* < 0.1, Wilcoxon rank-sum test) detected in the comparison of *responders* (*R*) and *non*-*responders* (*NR*). **c** CAZy class comparison between *R* and *NR*. *p < 0.1, **p < 0.05. **d** Performance of the C5.0 decision tree models in classifying *R* and *NR*

The Bray-Curtis dissimilarities based on 146 annotated KEGG pathway abundances illustrate the marginally separate clusters of *R* and *NR* (*p* = 0.0299, ANOSIM) (Fig. 3a). The KEGG pathway enrichment analysis of the metagenomic data shows that the majority of 32 pathways overrepresented in *NR* were catabolic pathways including ABC transporter, phosphotransferase system (PTS), carbohydrate metabolism pathways, and xenobiotic degradation pathways (FDR *p* < 0.1, Wilcoxon rank-sum test) (Fig. 3b), whereas anabolic pathways were in contrast overrepresented in *R*. This tendency is also consistent with the recently published study of anti-PD-1 immunotherapy in melanoma patients, which also reported that *NR* patients’ intestinal microbial communities had more enriched catabolic pathways compared to *R* [12]. Additionally, the Carbohydrate-Active enZymes (CAZy) annotation and the analysis of Clusters of Orthologous Groups (COG) supported the overrepresentation of catabolic functions in *NR*; three CAZy classes, “glycoside hydrolases,” “carbohydrate-binding modules,” and “auxiliary activities” were overrepresented in *NR* (FDR *p* < 0.1, Wilcoxon rank-sum test), whereas no CAZy classes were significantly enriched in *R* (FDR *p* > 0.1, Wilcoxon rank-sum test) (Fig. 3c); *NR* had six enriched COG classes including “carbohydrate transport and metabolism” and “amino acid transport and metabolism” (FDR *p* < 0.1, Wilcoxon rank-sum test) (Fig. S7). Although anabolic functions such as “valine, leucine, and isoleucine biosynthesis” and “unsaturated fatty acids biosynthesis” were exceptionally enriched in *NR*, these BCAA microbial metabolites have been found to be positively associated with cancers and related to tumor metabolic needs [27]. Likewise, unsaturated fatty acids have been suggested to be involved in the metastasis and stemness of certain cancers [28]. Furthermore, previous case-control gut microbiome studies reported that enrichment of ABC transporter and PTS in microbial communities are associated with inflammation, which has been shown to promote tumor growth in cancer patients [29].

In contrast, the pathway enrichment analysis revealed that the most significantly enriched pathways in *R* were biosynthetic pathways of metabolites including flavonoid, zeatin, and secondary bile acids (FDR *p* < 0.1, Wilcoxon rank-sum test) (Fig. 3b). The comparison of KEGG modules revealed that in *R*, 20 modules including the biosynthesis of lipopolysaccharide (LPS) were enriched (FDR *p* < 0.1, Wilcoxon rank-sum test) (Fig. S8). Bacterial LPS is known to induce the differentiation of Th17 cells [30].

### Initial microbiota composition and functionality predicts response to treatment

After identifying differences in intestinal microbial composition between *R* and *NR* in our cohort, we examined whether statistical modeling would enable prediction of treatment response based on the initial gut microbial status of the cancer patients. In addition to the anticancer therapy response, a recent study showed that the anti-integrin therapy response of inflammatory bowel disease patients could be predicted using the information of initial conditions of their preselected gut microbiota features based on a deep neural network [31]. However, to the best of our knowledge, there are no models used to predict the anticancer treatment response that covers broad types of cancer and treatments. We built a classification model based on decision tree using the features of baseline samples with a fivefold cross-validation. We used the relative abundances at the baseline of 31 differentially abundant species between *R* and *NR* (Fig. 1g) and the baseline RPKM of the differentially abundant KEGG pathways (Fig. 3b). The model performance was evaluated with an area under the curve (AUC) of receiver operating characteristic (ROC). Using the initial relative abundance of differentially abundant species solely, the performance was the lowest (AUC = 0.652) (Fig. 3d). The prediction performance was significantly improved by using the RPKM of differentially abundant KEGG pathways solely (AUC = 0.707). However, the model incorporating data on both species and pathways showed the best performance (AUC = 0.895), indicating the power of shotgun metagenomics for predicting host phenotypes. To further test the general applicability of the model, we recruited additional cancer patients and performed metagenomics sequencing in seven more patients (baseline samples from *R* = 5, *NR* = 2) to serve as an independent validation dataset. Using the initial relative abundance of differentially abundant species and the RPKM of differentially abundant KEGG pathways, we could achieve an AUC = 0.75.

The high accuracy of our prediction models indicates that the initial condition of the gut microbiota could be a potential predictive tool for response to anticancer treatments. Furthermore, the performance comparisons of our models suggest that combining the features of both taxa and functions improves the prediction accuracy.

### Oral gavage of responder bacteria reduces tumor size during erlotinib treatment in mice

**Fig. 4:**
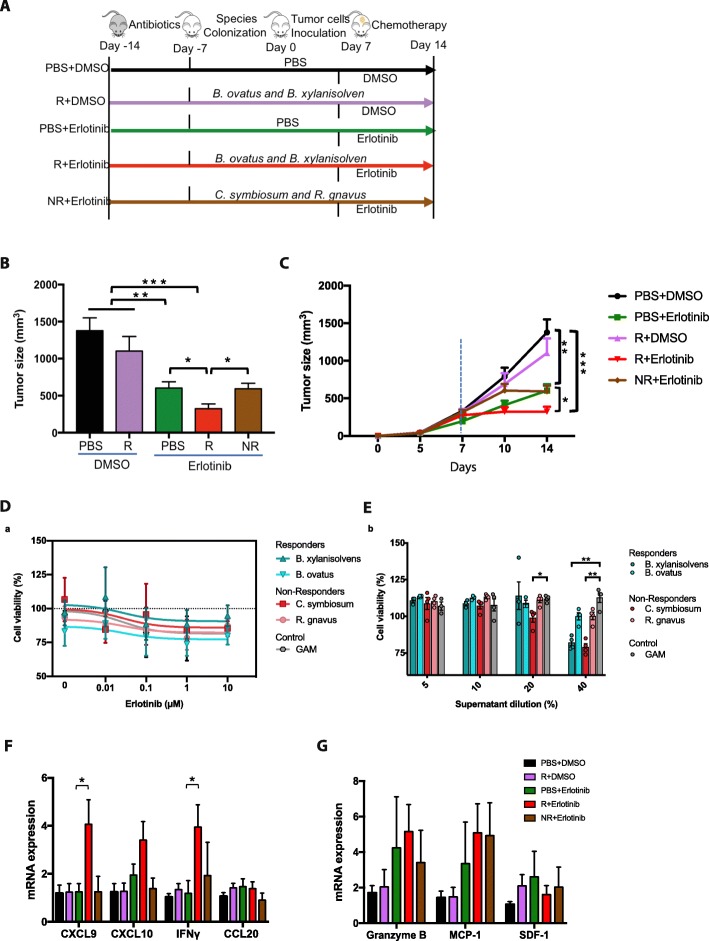
Increased anti-tumor efficacy of chemotherapy in the presence of *B*. *ovatus* and *B*. *xylanisolvens.***a** Experimental design: male 6-week C57BL6/N mice (*n* = 5–8) were treated with antibiotic cocktail in drinking water for 1 week before bacterial oral gavage. Control PBS, *B*. *ovatus* and *B*. *xylanisolvens*, and *C. symbiosum* and *R. gnavus* were orally gavaged into mice 1 week prior to tumor cell inoculation. A total of 10^7^ Lewis lung cancer cells in 200 μl PBS were subcutaneously injected into the mice to induce tumor formation. Mice were treated with erlotinib (60 mg/kg body weight) once the tumor size reached approximately 250–500 mm^3^. Time in days is relative to tumor cells injection. **b** Tumor size measurement at day 14. **c** Tumor growth curve after Lewis lung carcinoma cell inoculation. Dark dots indicate the application of erlotinib. **d**, **e** CRL5883 bronchoalveolar carcinoma cell line was cultured for 72 h in the presence of erlotinib (**d**) or drug-free (**e**) supernatants from *R* (*B.* xylanisolvens and *B. ovatus*) or *NR* (*R*. *gnavus* and *C*. *symbiosum*) bacteria species. **d** Non-linear regression curves showing cell viability as percentage of cell control viability. Bacterial supernatants had *n* = 4, GAM control had *n* = 2, and cell control had *n* = 10. **e** Cell viability is presented as percentage of cell control viability. Colored circles show individual data points. Outliers were identified and removed by the ROUT method (*Q* = 0.1%). Supernatants had *n* = 3–4 and cell control had *n* = 16. All data are mean ± SEM. Significant differences were identified via unpaired *t* test (*p < 0.05, **p < 0.005). **f**, **g** Tumor expressions of chemokines involved in the recruitment of T cells (**f**), myeloid cells, and cytotoxic T cells (**g**) by real-time PCR (normalized against *GAPDH*). Data are presented as mean ± SEM. *p < 0.05, **p < 0.01, ***p < 0.001

To test if there is a causal effect of the *R* and *NR* bacteria on treatment outcomes, we tested their impact on tumor growth in a murine lung cancer model [32]. As examples of the *R*-*enriched* bacteria, *B. ovatus* and *B. xylanisolvens* were chosen due to their relatively high significance in the species enrichment analysis described above (Fig. 1g). In addition, we selected *C*. *symbiosum* and *R*. *gnavus* due to their relatively high prevalence (63% and 67% for *C*. *symbiosum* and *R*. *gnavus*, respectively) in *NR* samples (Fig. S9). We selected Lewis lung carcinoma cells and erlotinib to test in the murine model, as the majority of our patient cohort suffered from forms of lung cancer, and erlotinib is a commonly used drug for non-small cell lung cancers [33]. We introduced either *R* (*B*. *ovatus* and *B*. *xylanisolvens*) or *NR* bacteria (*C*. *symbiosum* and *R*. *gnavus)* by daily oral gavage in antibiotic-pretreated mice (Fig. 4a and Fig. S10). One week later, Lewis lung carcinoma cells were subcutaneously inoculated into these C57BL/6 N mice to induce tumor formation. When the tumor size reached approximately 250–500 mm^3^, erlotinib was administered. Erlotinib significantly inhibited the tumor growth by 56% compared to the control group (PBS + DMSO) after 1 week (Fig. 4b and Fig. 4c). The *R*-*enriched* species alone reduced (by 20%) the tumor progression in mice compared to the control, but the difference was not statistically significant (*p* = 0.1949, Wilcoxon rank-sum test). However, the presence of *B*. *ovatus* and *B*. *xylanisolvens* led to additional significant reductions in tumor size in the erlotinib-treated mice (Fig. 4b). On day 14, the average tumor volume in erlotinib-treated mice colonized with the *R*-*enriched* species (*R* + erlotinib) was significantly smaller (46%) than that of the erlotinib-treated group (PBS + erlotinib) (*p* = 0.032, Wilcoxon rank-sum test), as well as that of the *NR* + erlotinib group (Fig. 4b and Fig. 4c) (*p* = 0.032, Wilcoxon rank-sum test). This demonstrates that simultaneous administration of *B*. *ovatus* and *B*. *xylanisolvens* increases the efficacy of erlotinib, suggesting that these *R*-*enriched* species could have a positive impact on therapeutic outcome in cancer. Interestingly, by comparing the tumor sizes among groups on day 10, the *NR* + erlotinib group had a significantly larger tumor size (87%) compared to that of *R* + erlotinib (*p* = 0.0317, Wilcoxon rank-sum test), which was commensurate with the control group without erlotinib (PBS + DMSO and *R* + DMSO) (Fig. 4c). This suggests the potential contribution of *C*. *symbiosum* and *R*. *gnavus* on treatment resistance.

To assess if there was a direct impact of the *R* bacteria on drug efficacy, we grew the *R* and *NR* bacteria in GAM media containing erlotinib. Subsequent addition of this spent media to the bronchoalveolar carcinoma cell line NCI-H1650 did not result in significant changes in the IC50 of erlotinib suggesting a limited direct impact of the *R* bacteria on erlotinib (Fig. 4d). To further investigate if metabolites produced by *R* and *NR* bacteria could directly affect the growth of cancer cells, we tested different dilutions of spent media from the *R* and *NR* bacteria on NCI-H1650 cell line viability. We observed that increasing amounts of spent media affected cancer cell line viability. The viability effects were species-specific and varied within the *R* and *NR* groups (Fig. 4e). These in vitro data suggest that bacterial effects on treatment outcome might be caused by multiple rather than single species acting in a consortium or that the beneficial effects depend on the host response to the specific bacteria.

To explore the mechanisms of how *R*-*enriched* bacteria increase the efficacy of chemotherapy, we examined the tumor expression of different chemokines involved in tumor progression using real-time PCR. Chemokines serve as attractant cytokines for different immune cells to modulate tumor growth through immunoediting. We found a significant increase in the expression of the chemokine (C-X-C motif) ligand 9 (CXCL9) and interferon gamma (IFN-γ) in the tumors of erlotinib-treated mice colonized with *R*-*enriched* species (*R* + erlotinib) compared to that of the control group (PBS + DMSO). CXCL10 expression in tumors also exhibited an increased trend in erlotinib-treated mice colonized with *R*-*enriched* species (*R* + erlotinib) (Fig. 4f). These molecules, which are involved in the recruitment of T cells, are negatively associated with tumor progression [34, 35] (Fig. S11). Importantly, such alterations were observed in neither the *R*-*enriched*-treated group (*R* + DMSO) nor the erlotinib-treated group (PBS + erlotinib), suggesting that the presence of *R*-*enriched* bacteria and erlotinib has a synergistic effect in modulating the immune responses of T cells in tumors. We did not observe such a synergistic effect in the expression of granzyme B, which is a serine protease in the granules of cytotoxic T cells (Fig. 4g). Furthermore, the levels of two chemokines, monocyte chemoattractant protein-1 (MCP-1) and stromal derived factor-1 (SDF-1), which are involved in the recruitment of myeloid cells, were comparable among these different groups (Fig. 4g). These findings suggest that the enhancement of chemotherapy efficacy by *R*-*enriched* bacteria may be achieved by synergistically upregulating the expression of chemokines involved in the recruitment of T cells.

## Discussion

We evaluated here for the first time the role of the gut microbiota in a heterogeneous patient cohort with various types of cancer and anticancer treatments to identify microbes with an impact on immune response. We identified significant differences in the gut microbiota composition and functional repertoire between *R* and *NR*, which were highly associated with treatment efficacy. Based on shotgun metagenomic data, we constructed and validated a statistical model that could predict cancer treatment outcomes with high accuracy in an independent validation cohort.

Despite the successful validation of the role of *R*-enriched bacteria in an animal model, our study comes also with limitations. First, while the response criteria were uniformly applied across treatment and cancer type as is typically performed in clinical trials, the likelihood of responsiveness may vary by line of therapy and clinical context. We focused on the microbiota signature that differentiated based on clinical outcome, not the cancer type or therapy. Second, due to a relatively small number of patients, we have also included a relatively small independent clinical cohort of patients for validation of the microbiota signature. A larger cohort will definitely provide the chance to overcome the issues with potential confounding factors and facilitate the detailed investigations into the effects of cancer types and treatments on gut microbiota. However, even with a small cohort, a solid conclusion and/or a highly accurate predictive model could be made from the comparison between groups in recent gut microbiome studies [36–38]. We also believe that mechanistic and biologic support for our findings from the clinical cohort was validated in the preclinical studies. Furthermore, future studies may investigate whether the NR-associated species can promote tumor growth and cancer progression in the absence of drug treatment, given the larger tumor size of *NR* + erlotinib group than the PBS + erlotinib group observed at day 10 (Fig. 4c). In addition, even though the functional analyses based solely on metagenomic data have shed lights on the potential mechanisms of gut microbiota affecting treatment outcomes, the use of metatranscriptomics and metabolomics to measure the actively expressed gut microbial functions and functional end-products, respectively, can lead to more robust and solid findings. Lastly, the murine experiment used erlotinib, an EGFR tyrosine kinase inhibitor, and not a cytotoxic chemotherapy. Typically, in current clinical practice, erlotinib is prescribed to advanced non-small cell lung cancer patients with tumors harboring an EGFR sensitizing mutation, due to its higher likelihood of response rate and lower overall toxicity rate relative to cytotoxic chemotherapy. However, the original U.S. Food and Drug Administration approval was based on response rate and non-small cell lung cancer, regardless of EGFR mutation status. Erlotinib was one of the treatments from the patient cohort. The use of single agent erlotinib in the murine experiment obviated the need to use potentially more confounding regimens to demonstrate the role of the microbiota such as doublet platinum-based chemotherapy or use of a single agent cytotoxic chemotherapy approved in NSCLC (docetaxel) that was not explored in the patient cohort and may have required additional optimal dose finding for these chemotherapeutics.

A recent study identified a consortium of 11 commensal bacterial species that were able to induce intestinal IFN-γ-producing CD8 T cells [15]. The investigators demonstrated that this bacterial consortium significantly enhanced efficacy of a checkpoint inhibitor treatment in a syngeneic mouse tumor model. We hypothesized that our identified *R* consortium could similarly activate cells of the immune system, which, in turn, would enhance the susceptibility of cancer cells to treatment outcome. Consequently, we found that the two species enriched in the *R* group, *B. xylanisolvens* and *B. ovatus*, in combination showed a synergistic effect with erlotinib. This effect on tumor progression could be partially mediated by activating the intratumoral mRNA expression of chemokines, which recruits dendritic cells and T cells. This observation is consistent with previous reports that indicate the infiltration of beneficial T cells into the intratumoral microenvironment mediated by specific gut bacteria, resulting in tumor size reduction. We previously revealed that a novel probiotics mixture can suppress hepatocellular carcinoma growth in mice by reducing the frequency of Th17 cells, the main producers of the IL-17 cytokine, in the intestine and their subsequent recruitment to the tumor bed [9], whereas *Akkermansia muciniphila* was recently identified as being associated with increased intratumoral immune infiltrates into the tumor bed in response to PD-1 blockade therapy [13]. Taken together, we believe that the administration of specific probiotic bacteria could be a potential supplemental treatment in combination with anticancer therapies for a better treatment outcome.

## Conclusions

The global cancer burden has risen dramatically making it an urgent need to develop novel therapies and predict which treatment will offer the most benefit to a cancer patient. Here, we analyzed the gut microbiota in a cohort that included eight different cancer types using metagenomic sequencing and found out that gut microbiome signatures at baseline accurately predict cancer treatment outcome. Furthermore, by evaluating the role of the gut microbiota for the first time in a heterogeneous patient cohort with various types of cancer and anticancer treatments, we have demonstrated a more global finding of a microbiota signature that is independent of cancer type and heterogeneity. Moreover, oral gavage of specific gut microbes significantly increased the effect of chemotherapy in mice, reducing the tumor volume by 46% compared to the control.

## Materials and methods

### Cancer cohort and treatment outcomes

The 26 cancer patients signed informed consent forms and were enrolled at the Western Regional Medical Center, Goodyear, AZ, after Western Institutional Review Board approval (WIRB #20140271). The patients were diagnosed with eight types of cancers and received either chemotherapy or a combination of chemo- and immunotherapy (Table S1). The 26 patients were classified into *responders* (*n* = 16) and *non-responders* (*n* = 10) based on their responses to anticancer treatment as defined by RECIST 1.1 [24] and irRECIST [25]. Furthermore, seven more additional cancer patients were recruited and metagenomics sequencing were performed to serve as an independent validation dataset (baseline samples from *R* = 5, *NR* = 2) to test the general applicability of the prediction model. The taxonomic profiles for a total of 138 stool samples from the Human Microbiome Project (HMP), as provided by MetaPhlAn2 [39] (http://segatalab.cibio.unitn.it/tools/metaphlan2/), were used as a healthy control in the taxa comparison.

### Metagenomic library construction and sequencing

To examine the gut microbiome of our cancer cohort, 71 fecal samples were collected longitudinally from 26 patients before and after treatments. Bacterial DNA was isolated from the fecal samples for shotgun metagenomic sequencing. Library preparation (using KAPA Hyper Prep Kit KR0961-V1.14) and Illumina sequencing were done at the University of Hong Kong, Centre for Genomic Sciences (HKU, CGS), using Illumina HiSeq 1500 with PE100 at an average depth of 6.1 Gbp (s.d. 1.3 Gbp per sample) (deposited in the European Nucleotide Archive with accession number PRJNA494824).

### Quality control and taxonomic profiling

The sequenced reads were processed with quality control to remove the adapter regions, low quality reads/bases using *fqc.pl* with default settings (https://github.com/TingtZHENG/VirMiner/tree/master/scripts/PipelineForQC) [40], and human DNA contaminations (bwa (version 0.7.4-r385) *mem* against human reference genome ucsc.hg19), following the previously described steps [9, 41]. Approximately 85% of the reads on average remained after the quality control and were used in downstream analyses. The high-quality reads were taxonomically profiled at different taxonomic levels using MetaPhlAn2 [39] with default settings, generating taxonomic relative abundances (total sum scaling normalization). The differentially abundant taxa were identified by the Wilcoxon rank-sum test, and the statistical significance was adjusted for multiple testing using FDR correction with the cutoff adjusted *p* value < 0.05, unless otherwise stated. ConStrains was utilized for strain level analysis with default settings [42].

### Microbial community diversity analysis

The alpha diversity (Shannon index) of each sample was calculated with R package VEGAN [43] (v2.5.3) on the relative abundance of species. Species richness for all samples were estimated based on rarefied data. Beta diversities (Bray-Curtis dissimilarities) among samples were calculated with VEGAN based on the relative abundance of species. To test the difference in the microbial composition between two or more groups, ANOSIM (analysis of similarities) was employed based on the Bray-Curtis dissimilarity.

### Species co-abundance network inference

For species co-abundance network reconstruction, the OTU relative abundance table was split into *responder* and *non-responder* samples, and they were processed independently with BAnOCC [26] for co-abundance network inference with 5000 iterations. A correlation estimate is considered significant if the corresponding 95% credible interval excludes zero. The estimated correlations were then filtered with the absolute values of correlation coefficients ≥ 0.4. The co-abundance network was visualized by Cytoscape 3.6.1. For visualizing Fig. 2, the subsets of networks were taken by extracting the edges that are connected with *B*. *ovatus*, *B*. *xylanisolvens*, *C*. *symbiosum*, and *R*. *gnavus*.

### De novo assembly and functional annotation

The high-quality reads after quality control were assembled using IDBA-UD [44] with k-mer size ranging from 20 to 100 bp. The coding DNA sequence (CDS) regions were predicted using MetaGeneMark [45] with the default parameters. The predicted peptide sequences were mapped to the KOBAS database [46] and dbCAN database [47] using DIAMOND [48] with the default parameters for KEGG (through KOBAS 2.0 *annotate* program) and CAZy annotation, respectively. The protein sequences were also assigned to the functional category of COG [49] using NCBI RPS-BLAST with default parameters. The abundance of genes was quantified in an RPKM (Reads Per Kilobase of transcript per Million mapped reads)-like manner using custom Perl scripts. Bray-Curtis dissimilarity calculated with VEGAN (v2.5.3) based on KEGG Orthologs was used to evaluate functional diversity between samples. KEGG pathway and module abundances were estimated by summing up the abundances of all genes present in the corresponding pathway or module (KEGG database accessed in December 2017).

### Classifier model

Fivefold cross-validation was performed on a C5.0 decision tree model (R 3.3.0, C50 0.1.1 package), using as predictors the differentially abundant species (FDR *p* < 0.05) and pathways (FDR *p* < 0.05) that were identified by comparing *responders* and *non-responders*. As a reference, we cited a study that used preselected features to build a classification model to predict the therapy response of inflammatory bowel disease [31].

### Bacterial strains and culture conditions

*Bacteroides ovatus* (ATCC 8483), *Bacteroides xylanisolvens* (DSM-18836), *Ruminococcus gnavus* (ATCC 29149), and *Clostridium symbiosum* (ATCC 14940) were grown at 37 °C under anaerobic conditions (Anaerobic gas mixture, 95% N_2_ and 5% H_2_) in pre-reduced GAM (Gifu anaerobic media; Nissui Pharmaceutical Co. Ltd.) broth for liquid culture or broth supplemented with agar (Gifu anaerobic media agar; Nissui Pharmaceutical Co. Ltd.) for growth on plates.

### Cell lines and culture conditions

The bronchoalveolar carcinoma cell line NCI-H1650 (ATCC CRL-5883) was cultured at 37 °C under 5% CO_2_ in Roswell Park Memorial Institute (RPMI) 1640 medium (ATCC modification; Thermo Fisher Scientific) supplemented with 10% Fetal Bovine Serum (FBS; Himedialabs) and antibiotics (~ 5000 units penicillin, 5 mg streptomycin, and 10 mg neomycin/mL). The cell line was maintained from frozen stock and allowed to grow for a minimum of 3 days before being used in the supernatant assays. Passage number was kept below 10. Lewis lung cancer cells (LLC) were cultured at 37 °C under 5% CO_2_ in Dulbecco’s modified Eagle medium (DMEM; Life technologies) supplemented with 10% FBS and antibiotics (100 U penicillin, 0.1 mg streptomycin, and 0.25 μg/ml amphotericin B).

### Supernatant exposure assay

Bacterial strains growing overnight in GAM broth were sub-cultured 1:50 in fresh GAM broth and grown for 24 h. Bacterial cultures were spun down at 11,000×*g* for 2 min and the supernatant carefully removed without disturbing the pellet. The supernatants were filtered through a 0.2-μM syringe filter to remove any remaining bacteria in suspension. For the erlotinib supernatant assay, 15 ml conical Greiner tubes (Sigma-Aldrich) were filled with GAM broth supplemented with an erlotinib (erlotinib hydrochloride dissolved in DMSO; Sigma-Aldrich) gradient ranging from 0 to 100 μM. The tubes were inoculated 1:50 with sub-cultured bacteria growing for 24 h. The bacterial culture was exposed to erlotinib for 24 h, before following the same procedure for supernatant preparation as described above. Supernatants were stored at − 20 °C until being un-thawed and homogenized by vortexing for the subsequent assays. Wells of a black, clear bottom 96-well plate were seeded with NCI-H1650 cells at a density of 5 × 10^3^ in either 90 μl or 50 μl of complete growth medium with antibiotics for the erlotinib or drug-free supernatant assays, respectively. Cells were allowed to attach for 1 day.

The following day, respective bacterial supernatants were added to the attached cells at a ratio of 1:10 or 1:1 for the erlotinib or drug-free supernatant assays, respectively. Dilution of supernatants resulted in final erlotinib concentrations of 0–10 μM and final supernatant dilutions of 0–40% in the respective wells. Cell control wells received either DMSO or PBS for the erlotinib or supernatant assay, respectively. GAM control wells were bacteria free and otherwise handled the same as bacterial supernatants. In both assays, plates were incubated for 72 h at 37 °C under 5% CO_2_. Viability was assessed by addition of 5% of a resazurin-based cell viability reagent (alamarBlue; Thermo Fisher Scientific) and further incubation for approximately 18 h. The reducing capability of viable cells was assessed by measuring fluorescence at 530EX nm/590EM nm in a Synergy H1 microplate reader (BioTek). Higher fluorescence signal indicated higher cell viability.

### Animal studies

Six-week old C57BL6/N mice were fed on a normal chow diet ad libitum. Mice were treated with a cocktail of antibiotics (ampicillin 0.3 g/L, neomycin 0.3 g/L, metronidazole 0.3 g/L, and vancomycin 0.15 g/L) in drinking water for 1 week before oral gavage of bacterial species. Control PBS, *responder*-*enriched* species (*B*. *ovatus* and *B. xylanisolvens*) and *non-responder*-*enriched* species (*C*. *symbiosum* and *R. gnavus*) were orally gavaged into mice respectively 1 week prior to the inoculation of the tumor cell line and daily throughout the entire experiments. To induce tumor formation, 10^7^ Lewis lung cancer cells in 200 μl PBS were subcutaneously injected into the mice. Mice were treated with or without erlotinib (60 mg/kg body weight) once the tumor size reached approximately 250–500 mm^3^. Tumor growth was assessed using a caliper, and tumor size was estimated by using the following formula: tumor size = length × width × width/2. All animal experiments were approved by the Committee on the Use of Live Animals for Teaching and Research of the University of Hong Kong.

### Gut colonization with responder-enriched species and non-responder-enriched species

*B*. *ovatus*, *B*. *xylanisolvens*, *C*. *symbiosum*, and *R. gnavus* were cultured anaerobically in GAM (Gifu anaerobic medium) broth. Colonization of antibiotic-pretreated C57BL/6 N mice was performed by oral gavage with 200 μl of suspension containing 5 × 10^9^ bacteria. The efficacy of colonization was confirmed by detecting the fecal content of bacterial species on day 14 (at the end of the experimental stage), based on pre-built standard curves and normalization by the gram of feces. Fecal DNA was extracted with the QIAamp DNA stool mini kit (Qiagen) and subjected to PCR amplification targeting different bacterial species. Primers for *B*. *ovatus* and *B*. *xylanisolvens* were as follows: forward: GGTGTCGGCTTAAGTGCCAT; reverse: CGGACGTAAGGGCCGTGC. Primers for *C*. *symbiosum* and *R*. *gnavus* were as follows: forward: CGGTACCTGACTAAGAAGC; reverse: AGTTTCATTCTTGCGAACG.

### Quantitative real-time PCR

Tumors were frozen in liquid nitrogen immediately after harvest, and total RNA was extracted with RNAiso Plus (Takara) and reverse transcribed into complementary DNA with a primeScript RT reagent kit (Takara). Quantitative real-time PCR was performed by using SYBR Premix Ex Taq (Takara) with specific primers on a StepOnePlus Real-time PCR system (Applied Biosystems). Primers for *CXCL9* were as follows: forward: GGAGTTCGAGGAACCCTAGTG; reverse: GGGATTTGTAGTGGATCGTGC. Primers for *CXCL10* were as follows: forward: CCAAGTGCTGCCGTCATTTTC; reverse: TCCCTATGGCCCTCATTCTCA. Primers for *IFN-*γ were as follows: forward: ATGAACGCTACACACTGCATC; reverse: CCATCCTTTTGCCAGTTCCTC. Primers for *CCL20* were as follows: forward: ACTGTTGCCTCTCGTACATACA; reverse: GAGGAGGTTCACAGCCCTTTT. Primers for *granzyme B* were as follows: forward: TCTCGACCCTACATGGCCTTA; reverse: TCCTGTTCTTTGATGTTGTGGG. Primers for *MCP*-*1* were as follows: forward: CCACTCACCTGCTGCTACTCA; reverse: TGGTGATCCTCTTGTAGCTCTCC. Primers for *SDF*-*1* were as follows: forward: TGCATCAGTGACGGTAAACCA; reverse: CACAGTTTGGAGTGTTGAGGAT.

### Statistical analysis

'The significance of the differences between groups was analyzed using the Wilcoxon rank-sum test and ANOSIM with R. A *p* value < 0.05 (5% level of probability) was considered to be significant and denoted as follows: *p < 0.05, **p < 0.01, ***p < 0.001, and ****p < 0.0001. In in vitro assays, cell viability percentage was calculated as percentage of average viability from cell control wells. Outliers were identified with the ROUT method using a strict threshold of *Q* = 0.1%. Identified outliers were removed for subsequent statistical analysis. For non-linear regression curves, differences in IC50 values were determined with the extra sum-of-squares *F*-test. Significant differences between bacterial and GAM control wells were determined via an unpaired *t* test and a false discovery rate approach using the two-stage linear step-up procedure of Benjamini, Krieger, and Yekutieli, with a false discovery rate (*Q*) of 1%. Testing conditions were analyzed individually, without assuming a consistent SD. Statistical analysis in vitro was performed with GraphPad Prism (version 8.0.0 for Mac, GraphPad Software, San Diego, CA, USA, www.graphpad.com).

## Supplementary information

**Additional file 1: Fig. S1.** NMDS plot based on the gut microbial compositions at species level of cancer patients. (A) Intra-patient samples clustered together. (B) Baseline samples and Treatment samples (*p* = 0.364, ANOSIM). **Fig. S2.** Alpha-diversity comparison between Baseline (red) and Treatment (blue). **Fig. S3.** Comparison between cancer patient samples and Human Microbiome Project (HMP). (A) NMDS plot of cancer patient samples and HMP samples based on the gut microbial compositions at species level (*p* = 0.0001, ANOSIM). (B) Alpha-diversity comparison (*p* = 0.07373, Wilcoxon rank sum test). (C) Comparison between *Firmicutes*/*Bacteroidetes* ratio (*p* = 2.461e-13, Wilcoxon rank sum test). **Fig. S4.** Comparison of Species richness between *R* and *NR* samples. (A) Rarefaction curves of *R* and *NR* samples. (B) Comparison of Chao1 index between *R* and *NR* (*p* = 0.674, Wilcoxon rank sum test). **Fig. S5.** Treatment impacts measured based on the Bray-Curtis distance between baseline and treatment at (A) species level (*p* = 0.216, Wilcoxon rank sum test) and at (B) strain level (*p* = 0.204, Wilcoxon rank sum test). **Fig. S6.** Heatmap with Pearson correlation result between species relative abundances and *Firmicutes*/*Bacteroidetes* ratio in *NR* group. *p < 0.05. **Fig. S7.** Comparison of COG families between *R* and *NR*. *p < 0.1, **p < 0.05. **Fig. S8.***R*-enriched KEGG modules (FDR *p* < 0.1) detected in the comparison of *R* and *NR*. **Fig. S9.** Comparison of relative abundance (%) of *Clostridium Symbiosum* and *Ruminococcus gnavus* in *R* (blue) and *NR* (pink). **Fig. S10.** Colonization of (A) *R*-enriched and (B) *NR*-enriched species in mice. *B. ovatus* and *B. xylanisolvens* belong to *Bacteroides* group, and *C. symbiosum* and *R. gnavus* belongs to *C. coccoides*-*E. rectale* group. T-test: *p < 0.05, **p < 0.01, ***p < 0.001. **Fig. S11.** Scatter plots of Spearman’s rank correlation analysis results between the mRNA expression of chemokine and tumor size. **Table S1.** Patient information. **Table S2.** Summary of metagenomic sequencing data. Table S3. Baseline characteristics of cancer patients in this study cohort.

## Data Availability

The shotgun metagenomic sequences have been deposited in the European Nucleotide Archive under accession number PRJNA494824.
