# Supplementary material for: Predictable modulation of cancer treatment outcomes by the gut microbiota
Source: Microbiome. 2020 Mar 5;8:28. doi: 10.1186/s40168-020-00811-2 (PMC7059390; doi:10.1186/s40168-020-00811-2)
Supplement: Supplementary file 1 — Additional file 1: Fig. S1. NMDS plot based on the gut microbial compositions at species level of cancer patients. (A) Intra-patient samples clustered together. (B) Baseline samples and Treatment samples (p = 0.364, ANOSIM). Fig. S2. Alpha-diversity comparison between Baseline (red) and Treatment (blue). Fig. S3. Comparison between cancer patient samples and Human Microbiome Project (HMP). (A) NMDS plot of cancer patient samples and HMP samples based on the gut microbial compositions at species level (p = 0.0001, ANOSIM). (B) Alpha-diversity comparison (p = 0.07373, Wilcoxon rank sum test). (C) Comparison between Firmicutes/Bacteroidetes ratio (p = 2.461e-13, Wilcoxon rank sum test). Fig. S4. Comparison of Species richness between R and NR samples. (A) Rarefaction curves of R and NR samples. (B) Comparison of Chao1 index between R and NR (p = 0.674, Wilcoxon rank sum test). Fig. S5. Treatment impacts measured based on the Bray-Curtis distance between baseline and treatment at (A) species level (p = 0.216, Wilcoxon rank sum test) and at (B) strain level (p = 0.204, Wilcoxon rank sum test). Fig. S6. Heatmap with Pearson correlation result between species relative abundances and Firmicutes/Bacteroidetes ratio in NR group. *p < 0.05. Fig. S7. Comparison of COG families between R and NR. *p < 0.1, **p < 0.05. Fig. S8.R-enriched KEGG modules (FDR p < 0.1) detected in the comparison of R and NR. Fig. S9. Comparison of relative abundance (%) of Clostridium Symbiosum and Ruminococcus gnavus in R (blue) and NR (pink). Fig. S10. Colonization of (A) R-enriched and (B) NR-enriched species in mice. B. ovatus and B. xylanisolvens belong to Bacteroides group, and C. symbiosum and R. gnavus belongs to C. coccoides-E. rectale group. T-test: *p < 0.05, **p < 0.01, ***p < 0.001. Fig. S11. Scatter plots of Spearman’s rank correlation analysis results between the mRNA expression of chemokine and tumor size. Table S1. Patient information. Table S2. Summary of metagenomic sequencing data. [file 40168_2020_811_MOESM1_ESM.docx]

Supplementary Information

Predictable modulation of cancer treatment outcomes by the gut microbiota

Yoshitaro Heshiki^†^, Ruben Vazquez-Uribe^†^, Jin Li^†^, Yueqiong Ni^†^, Scott Quainoo, Lejla Imamovic, Jun Li, Maria Sørensen, Billy K. C. Chow, Glen J. Weiss^*^, Aimin Xu^*^, Morten O. A. Sommer^*^, and Gianni Panagiotou^*^

†These authors contributed equally to this work.

*Corresponding authors: Glen J. Weiss, Aimin Xu, Morten O. A. Sommer, and Gianni Panagiotou

Email: drglenweiss@outlook.com (GJW); [amxu@hku.hk](mailto:amxu@hku.hk) (AX); [msom@bio.dtu.dk](mailto:msom@bio.dtu.dk) (MOAS); [Gianni.Panagiotou@hki-jena.de](mailto:Gianni.Panagiotou@hki-jena.de) (GP)

**This PDF file includes:**

Figs. S1 to S11

Tables S1 to S3

**Fig. S1.** NMDS plot based on the gut microbial compositions at species level of cancer patients. (A) Intra-patient samples clustered together. (B) Baseline samples and Treatment samples (p=0.364, ANOSIM).


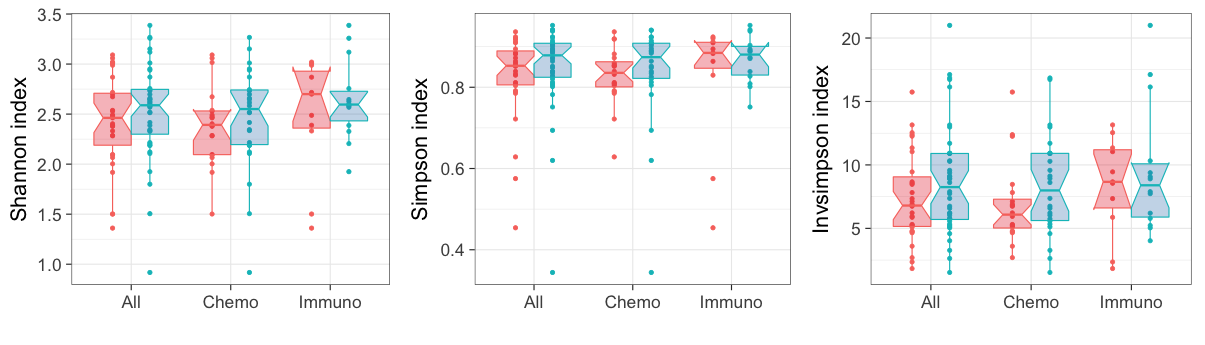
**Fig. S2.** Alpha-diversity comparison between Baseline (red) and Treatment (blue).

**Fig. S3.** Comparison between cancer patient samples and Human Microbiome Project (HMP). (A) NMDS plot of cancer patient samples and HMP samples based on the gut microbial compositions at species level (p=0.0001, ANOSIM). (B) Alpha-diversity comparison (p=0.07373, Wilcoxon rank sum test). (C) Comparison between *Firmicutes/Bacteroidetes* ratio (p=2.461e-13, Wilcoxon rank sum test).


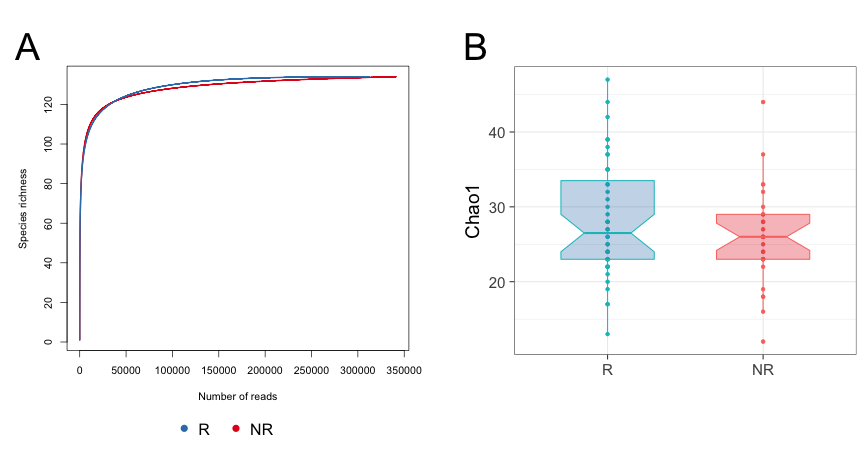


**Fig. S4.** Comparison of Species richness between *R* and *NR* samples. (A) Rarefaction curves of *R* and *NR* samples. (B) Comparison of Chao1 index between *R* and *NR* (p=0.674, Wilcoxon rank sum test).


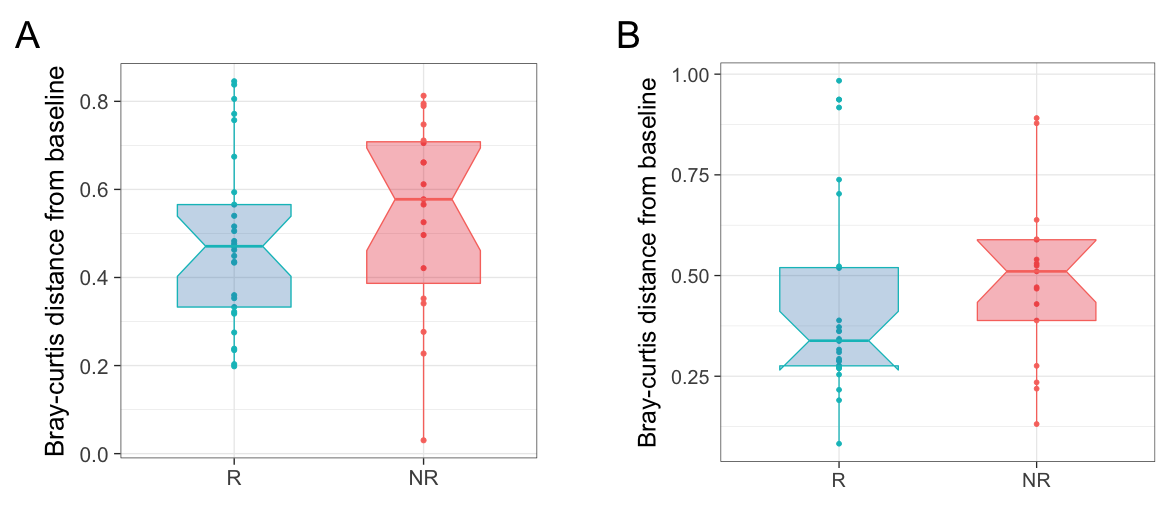
**Fig. S5.** Treatment impacts measured based on the Bray-Curtis distance between baseline and treatment at (A) species level (p=0.216, Wilcoxon rank sum test) and at (B) strain level (p=0.204, Wilcoxon rank sum test).

**Fig. S6.** Heatmap with Pearson correlation result between species relative abundances and *Firmicutes*/*Bacteroidetes* ratio in *NR* group. *p<0.05.

**Fig. S7.** Comparison of COG families between R and NR. *p< 0.1, **p<0.05.


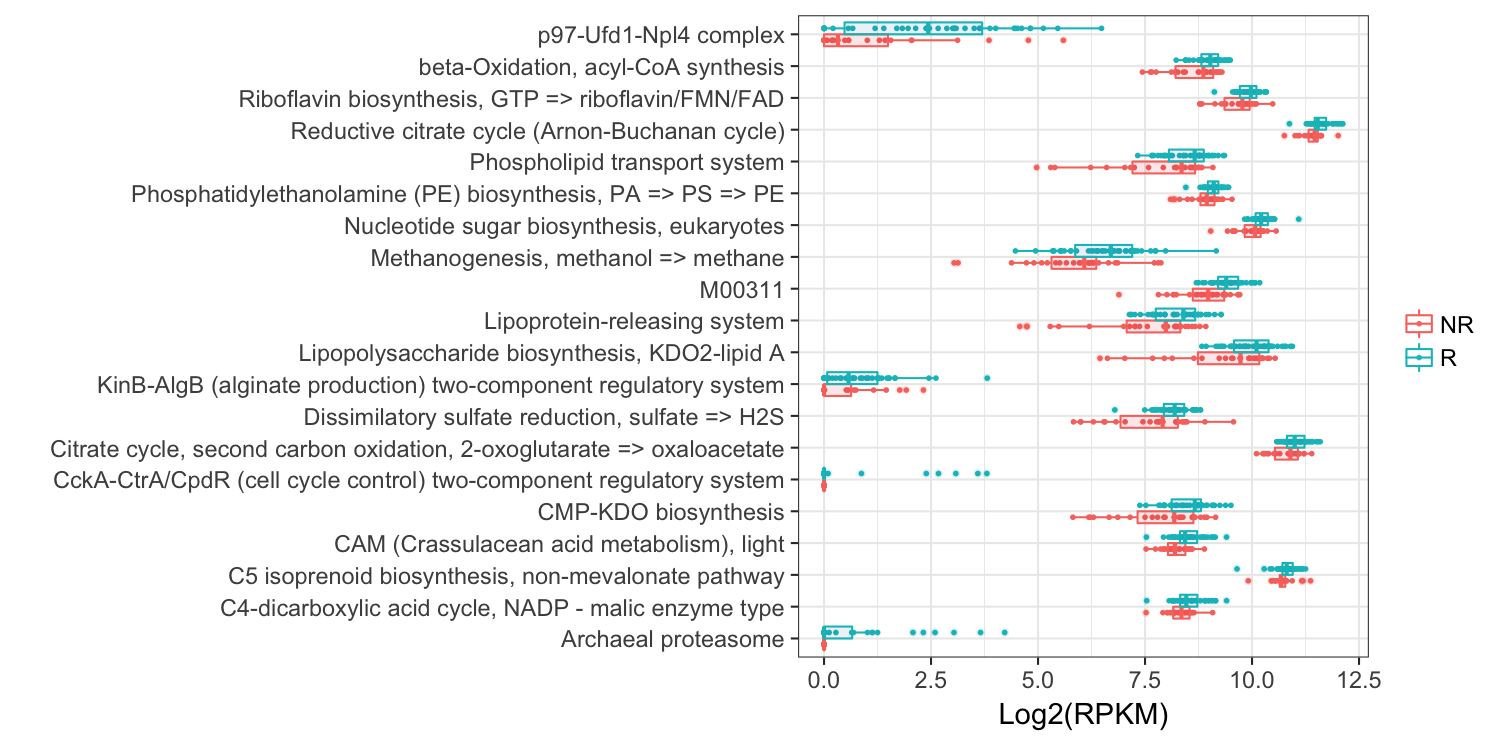
**Fig. S8.** *R-enriched* KEGG modules (FDR p<0.1) detected in the comparison of *R* and *NR*.


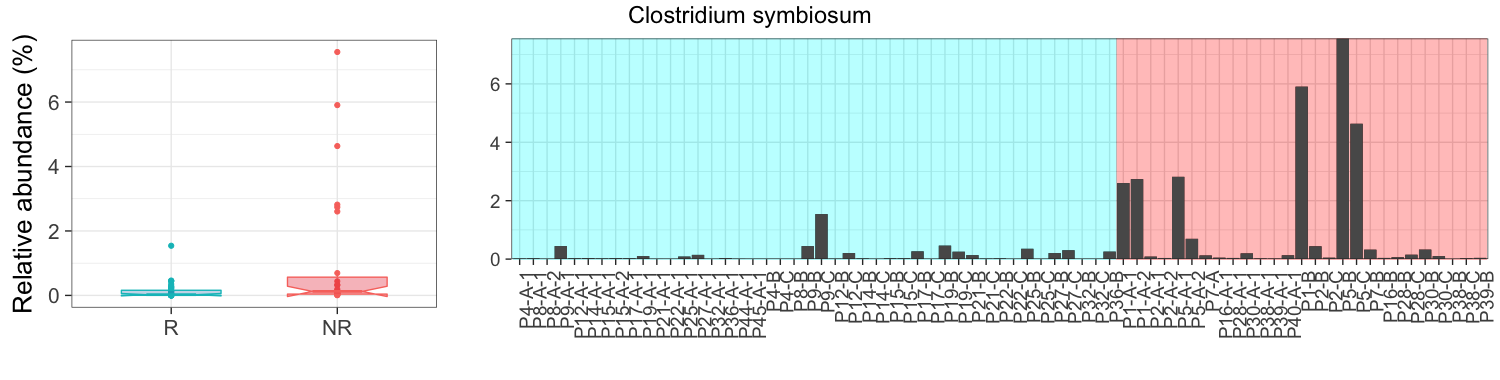

**Fig. S9.** Comparison of relative abundance (%) of *Clostridium Symbiosum* and *Ruminococcus gnavus* in *Responders* (blue) and *Non-Responders* (pink).

**Fig. S10.** Colonization of (A) responder-enriched and (B) non-responder-enriched species in mice. *B. ovatus* and *B. xylanisolvens* belong to *Bacteroides* group, and *C. symbiosum* and *R. gnavus* belongs to *C. coccoides*-*E. rectale* group. T-test: *p<0.05, **p<0.01, ***p<0.001.


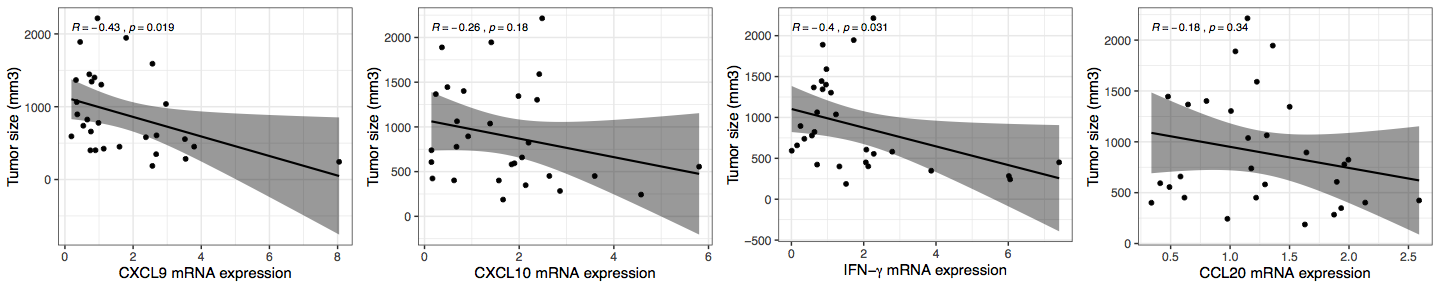

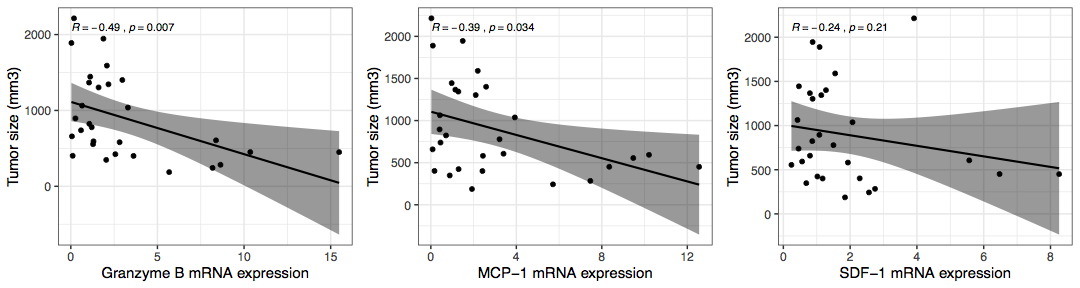


**Fig. S11.** Scatter plots of Spearman’s rank correlation analysis results between the mRNA expression of chemokine and tumor size.

**Table S1.** Patient information.

| ID | Age | Sex | Race | Cancer Type | Stage | Prior Treatment^#^ | Treatment | Clinical  Response |
| --- | --- | --- | --- | --- | --- | --- | --- | --- |
| 1 | 53 | F | Black | Lung adenocarcinoma | IV | Yes | Irinotecan | NR |
| 2 | 60 | F | Caucasian | Breast cancer | III | Yes | Everolimus | NR |
| 4 | 41 | M | Caucasian | Lung adenocarcinoma | IV | Not Available | Crizotinib | R |
| 5 | 54 | F | Caucasian | Lung adenocarcinoma | IV | Not Available | Erlotinib | NR |
| 7 | 53 | M | Caucasian | Lung adenocarcinoma | IV | Not Available | Crizotinib | NR |
| 8 | 51 | F | Caucasian | Lung adenocarcinoma | IV | Not Available | Erlotinib | R |
| 9 | 52 | F | Caucasian | Lung adenocarcinoma | IV | Not Available | Erlotinib | R |
| 12 | 64 | M | Hispanic | Lung adenocarcinoma | IV | Yes | Irinotecan | R |
| 14 | 58 | F | Caucasian | Breast cancer | I | Not Available | Letrozole | R |
| 15 | 64 | M | Caucasian | Prostate adenocarcinoma | IV | No | Enzalutimide | R |
| 16 | 64 | M | Hispanic | Prostate adenocarcinoma | IV | No | Enzalutimide | NR |
| 17 | 61 | F | Caucasian | Rectal adenocarcinoma | IIA | Yes | Capecitabine | R |
| 19 | 55 | M | Caucasian | Rectal adenocarcinoma | IVA | Yes | Capecitabine | R |
| 21 | 35 | M | Hispanic | Chronic myelogenous leukemia | N/A | No | Dasatinib | R |
| 22 | 64 | F | Caucasian | Breast cancer | IA | Not Available | Anastrazole | R |
| 25 | 60 | M | Caucasian | Small cell lung cancer | IV | Yes | Irinotecan/pembrolizumab | R |
| 27 | 72 | M | Caucasian | Colon adenocarcinoma | IV | Yes | Capecitabine/irinotecan/nivolumab | R |
| 28 | 58 | F | Caucasian | Breast cancer | IV | Yes | Gemcitabine/vinorelbine/pembrolizumab | NR |
| 30 | 43 | F | Black | Ovarian cancer | IV | Yes | Liposomal doxorubicin/pembrolizumab | NR |
| 32 | 51 | M | Black | Colon adenocarcinoma | IV | Yes | Capecitabine/irinotecan/nivolumab | R |
| 36 | 46 | F | Caucasian | Breast cancer | IV | Yes | Gemcitabine/vinorelbine/pembrolizumab | R |
| 38 | 42 | F | Caucasian | Breast cancer | IV | Yes | Ado-trastuzumab/pembrolizumab | NR |
| 39 | 54 | M | Caucasian | Pancreatic adenocarcinoma | IV | Yes | Capecitabine/irinotecan/nivolumab | NR |
| 40 | 54 | F | Caucasian | Pancreatic adenocarcinoma | IV | Yes | Capecitabine/irinotecan/nivolumab | NR |
| 44 | 36 | F | Asian | Breast cancer | IV | Yes | Trastuzumab/pembrolizumab | R |
| 45 | 38 | F | Caucasian | Ovarian cancer | IV | Yes | Liposomal doxorubicin/pembrolizumab | R |

# yes = prior systemic therapy (cytotoxic chemotherapy, targeted therapy, endocrine, or immunotherapy); no = no prior systemic therapy; not available = data on prior therapy could not be confirmed.

**Table S2.** Summary of metagenomic sequencing data.

| **Patient ID** | **Sample ID** | **Sample info** | **# of raw reads** | **# of raw base pairs** | **# of final usable reads** | **% of final usable reads** |
| --- | --- | --- | --- | --- | --- | --- |
| 1 | 1A1 | Baseline 1 | 51033598 | 7655039700 | 50347551 | 98.66% |
|  | 1A2 | Baseline 2 | 54348818 | 8152322700 | 53645665 | 98.71% |
|  | 1B | Cycle 1 | 51290529 | 7693579350 | 48077224 | 93.74% |
| 2 | 2A1 | Baseline 1 | 54618573 | 8192785950 | 53695238 | 98.31% |
|  | 2A2 | Baseline 2 | 52767405 | 7915110750 | 52165865 | 98.86% |
|  | 2B | Cycle 1 | 51567488 | 7735123200 | 50844494 | 98.60% |
|  | 2C | Cycle 2 | 52741023 | 7911153450 | 52134191 | 98.85% |
| 4 | 4A1 | Baseline 1 | 57558069 | 8633710350 | 56984827 | 99.00% |
|  | 4B | Cycle 1 | 48084771 | 7212715650 | 47386706 | 98.55% |
|  | 4C | Cycle 2 | 49500387 | 7425058050 | 49083325 | 99.16% |
| 5 | 5A1 | Baseline 1 | 45249727 | 6787459050 | 44863205 | 99.15% |
|  | 5A2 | Baseline 2 | 51288754 | 7693313100 | 50608804 | 98.67% |
|  | 5B | Cycle 1 | 53276018 | 7991402700 | 52736077 | 98.99% |
|  | 5C | Cycle 2 | 54636135 | 8195420250 | 53912605 | 98.68% |
| 7 | 7A | Baseline 1 | 49336862 | 7400529300 | 48125581 | 97.54% |
|  | 7B | Cycle 1 | 51786243 | 7767936450 | 50639593 | 97.79% |
| 8 | 8A1 | Baseline 1 | 49127892 | 7369183800 | 48704689 | 99.14% |
|  | 8A2 | Baseline 2 | 53363540 | 8004531000 | 52595667 | 98.56% |
|  | 8B | Cycle 1 | 50417760 | 7562664000 | 49930382 | 99.03% |
| 9 | 9A1 | Baseline 1 | 53415210 | 8012281500 | 51493345 | 96.40% |
|  | 9B | Cycle 1 | 52601182 | 7890177300 | 52089802 | 99.03% |
|  | 9C | Cycle 2 | 49268216 | 7390232400 | 48580451 | 98.60% |
| 12 | 12A1 | Baseline 1 | 54331254 | 8149688100 | 53865788 | 99.14% |
|  | 12B | Cycle 1 | 51928084 | 7789212600 | 51193278 | 98.58% |
|  | 12C | Cycle 2 | 53433742 | 8015061300 | 52505800 | 98.26% |
| 14 | 14A1 | Baseline 1 | 33909782 | 5086467300 | 33334668 | 98.30% |
|  | 14B | Cycle 1 | 30082507 | 4512376050 | 29778801 | 98.99% |
|  | 14C | Cycle 2 | 29736718 | 4460507700 | 29431650 | 98.97% |
| 15 | 15A1 | Baseline 1 | 27918738 | 4187810700 | 27557003 | 98.70% |
|  | 15A2 | Baseline 2 | 35727343 | 5359101450 | 35223831 | 98.59% |
|  | 15B | Cycle 1 | 32732155 | 4909823250 | 32356751 | 98.85% |
|  | 15C | Cycle 2 | 32506387 | 4875958050 | 32153083 | 98.91% |
| 16 | 16A1 | Baseline 1 | 29733351 | 4460002650 | 29428109 | 98.97% |
|  | 16B | Cycle 1 | 29114386 | 4367157900 | 28798933 | 98.92% |
| 17 | 17A1 | Baseline 1 | 35367275 | 5305091250 | 34367983 | 97.17% |
|  | 17B | Cycle 1 | 35025798 | 5253869700 | 34051556 | 97.22% |
|  | 17C | Cycle 2 | 35011475 | 5251721250 | 22205004 | 63.42% |
| 19 | 19A1 | Baseline 1 | 32095784 | 4814367600 | 5208242 | 16.23% |
|  | 19B | Cycle 1 | 34981049 | 5247157350 | 33341659 | 95.31% |
|  | 19C | Cycle 2 | 35826175 | 5373926250 | 35095619 | 97.96% |
| 21 | 21A1 | Baseline 1 | 36103991 | 5415598650 | 34472058 | 95.48% |
|  | 21B | Cycle 1 | 35457023 | 5318553450 | 34021930 | 95.95% |
|  | 21C | Cycle 2 | 34501967 | 5175295050 | 30760095 | 89.15% |
| 22 | 22A1 | Baseline 1 | 35660742 | 5349111300 | 34550907 | 96.89% |
|  | 22B | Cycle 1 | 38824174 | 5823626100 | 37826885 | 97.43% |
|  | 22C | Cycle 2 | 36968929 | 5545339350 | 32748176 | 88.58% |
| 25 | 25A1 | Baseline 1 | 34824150 | 5223622500 | 34195737 | 98.20% |
|  | 25B | Cycle 1 | 35102501 | 5265375150 | 32546942 | 92.72% |
|  | 25C | Cycle 2 | 35255929 | 5288389350 | 34568721 | 98.05% |
| 27 | 27A1 | Baseline 1 | 35986831 | 5398024650 | 35226731 | 97.89% |
|  | 27B | Cycle 1 | 36268429 | 5440264350 | 35499176 | 97.88% |
|  | 27C | Cycle 2 | 34620298 | 5193044700 | 33082978 | 95.56% |
| 28 | 28A1 | Baseline 1 | 37229856 | 5584478400 | 36469398 | 97.96% |
|  | 28B | Cycle 1 | 35711683 | 5356752450 | 33473805 | 93.73% |
|  | 28C | Cycle 2 | 36179842 | 5426976300 | 33507232 | 92.61% |
| 30 | 30A1 | Baseline 1 | 37593714 | 5639057100 | 36639121 | 97.46% |
|  | 30B | Cycle 1 | 37070989 | 5560648350 | 36233009 | 97.74% |
|  | 30C | Cycle 2 | 36220308 | 5433046200 | 33468899 | 92.40% |
| 32 | 32A1 | Baseline 1 | 35879553 | 5381932950 | 35107040 | 97.85% |
|  | 32B | Cycle 1 | 35095260 | 5264289000 | 34480409 | 98.25% |
|  | 32C | Cycle 2 | 35871328 | 5380699200 | 35131689 | 97.94% |
| 36 | 36A1 | Baseline 1 | 37198678 | 5579801700 | 36466650 | 98.03% |
|  | 36B | Cycle 1 | 34769792 | 5215468800 | 33582626 | 96.59% |
| 38 | 38A1 | Baseline 1 | 35805816 | 5370872400 | 35153781 | 98.18% |
|  | 38B | Cycle 1 | 36127351 | 5419102650 | 35474837 | 98.19% |
|  | 38C | Cycle 2 | 34621914 | 5193287100 | 33285628 | 96.14% |
| 39 | 39A1 | Baseline 1 | 37044874 | 5556731100 | 36319969 | 98.04% |
|  | 39B | Cycle 1 | 31414326 | 4712148900 | 27363654 | 87.11% |
| 40 | 40A1 | Baseline 1 | 35543044 | 5331456600 | 34471983 | 96.99% |
| 44 | 44A1 | Baseline 1 | 33580773 | 5037115950 | 32892581 | 97.95% |
| 45 | 45A1 | Baseline 1 | 35582940 | 5337441000 | 34870679 | 98.00% |

**Table S3.** Baseline characteristics of cancer patients in this study cohort.

|  | Responders  (n=16) | Non-responders  (n=10) | P-value | | Used test |
| --- | --- | --- | --- | --- | --- |
| **Age** |  |  | 0.9578 | Wilcoxon rank sum | |
| Median | 53.5 | 54 |  |  | |
| Range | 35-72 | 42-64 |  |  | |
| **Gender** |  |  | 0.427863777 | Fisher's exact | |
| Male | 8 | 3 |  |  | |
| Female | 8 | 7 |  |  | |
| **Prior Treatment^#^** |  |  | 0.84170144 | Fisher's exact | |
| Yes | 9 | 7 |  |  | |
| No | 2 | 1 |  |  | |
| Not Available | 5 | 2 |  |  | |
| **Therapy** |  |  | 0.689056401 | Fisher's exact | |
| Chemotherapy | 10 | 5 |  |  | |
| Immunotherapy | 6 | 5 |  |  | |
| **Race** |  |  | 0.871936759 | Fisher's exact | |
| White | 12 | 7 |  |  | |
| Black | 1 | 2 |  |  | |
| Hispanic | 2 | 1 |  |  | |
| Asian | 1 | 0 |  |  | |
| **Organ** |  |  | 0.658922744 | Fisher's exact | |
| Lung | 5 | 3 |  |  | |
| Breast | 4 | 3 |  |  | |
| Colon | 2 | 0 |  |  | |
| Rectal | 2 | 0 |  |  | |
| Ovarian | 1 | 1 |  |  | |
| Prostate | 1 | 1 |  |  | |
| Pancreatic | 0 | 2 |  |  | |
| Leukemia | 1 | 0 |  |  | |
| **Stage** |  |  | 0.965217391 | Fisher's exact | |
| I | 1 | 0 |  |  | |
| IA | 1 | 0 |  |  | |
| IIA | 1 | 0 |  |  | |
| III | 0 | 1 |  |  | |
| IV | 11 | 9 |  |  | |
| IVA | 1 | 0 |  |  | |
| NA | 1 | 0 |  |  | |

# yes = prior systemic therapy (cytotoxic chemotherapy, targeted therapy, endocrine, or immunotherapy); no = no prior systemic therapy; not available = data on prior therapy could not be confirmed.
